# Supplementary material for: A mode-locked random laser generating transform-limited optical pulses
Source: Nat Commun. 2024 Jan 2;15:177. doi: 10.1038/s41467-023-44315-7 (PMC10764872; doi:10.1038/s41467-023-44315-7)
Supplement: Supplementary file 1 — Supplementary Information [file 41467_2023_44315_MOESM1_ESM.pdf]

Supplementary information

Supplementary Methods

The fibre laser cavity used in this work consisted of a loop and an open-ended section as in Supplementary Figure 1. Gated amplification was provided inside the loop by driving with current pulses a semiconductor optical amplifier, Thorlabs SOA 1013SXS. A 125-m delay fibre was also included in the loop to increase the round-trip time of the cavity to the ~1 µs range and ensure that the cavity round trip for the farthest addressable section, at the end of the fibre, is less than twice the round trip for the closest addressable fibre section. In some experiments, particularly with very short-duration pulses, a commercial erbium-doped fibre amplifier (EDFA Thorlabs 100S) was also inserted in the loop. It amplified the weak backscattered signal from the distributed mirror by up to ~36 dB before the SOA. When the extra EDFA was included, an adjustable spectral filter (Alnair-Labs-CVF-300CL) was also used to reduce the amplified spontaneous emission and limit the feedback signal to the neighbourhood (<20 GHz) of the wavelength of interest. The 3 dB fused fibre coupler connected the loop to the open-ended fibre section of the cavity. A large fraction of the amplification needed for the laser to go above threshold (54.5 dB) was provided by a 27 m long Corning erbium-doped fibre (1550C3) without isolators, working in both directions. Gain was provided to the EDF by a 365 mW CW-pumped diode laser at 976 nm wavelength. An open-ended 63-m long spool of single-mode fibre completed the laser cavity. The output of the laser was monitored with a 26 GHz amplified photodiode model UPD-15-IR2-FC and a 3.5 GHz 40 GSamples/s Tektronix DPO7354 oscilloscope. The spectrum of the laser output was measured with an Apex AP2050A optical spectrum analyser with an optical frequency resolution as narrow as 20 MHz (0.16 pm wavelength). The current pulses for the SOA were provided by an Avtech pulse generator (AVM-2) delivering up to 300 milliamperes with a rise time <1ns and durations up to 20 ns. The trigger for the pulse generator came from a two channel Keysight arbitrary function generator Model 33500B which allowed for pulse frequency tuning to the millihertz resolution. The short section of fibre providing for Rayleigh backscattering was chosen in position and in length along the fibre spool by adjusting the repetition rate and the duration of the electrical pulses driving the SOA. The single-mode fibre end was angle-cleaved and bent sharply to prevent backscattering from the termination. Lasing was observed along the entire spool length, but the pulse amplitude and stability varied because of the random feedback.

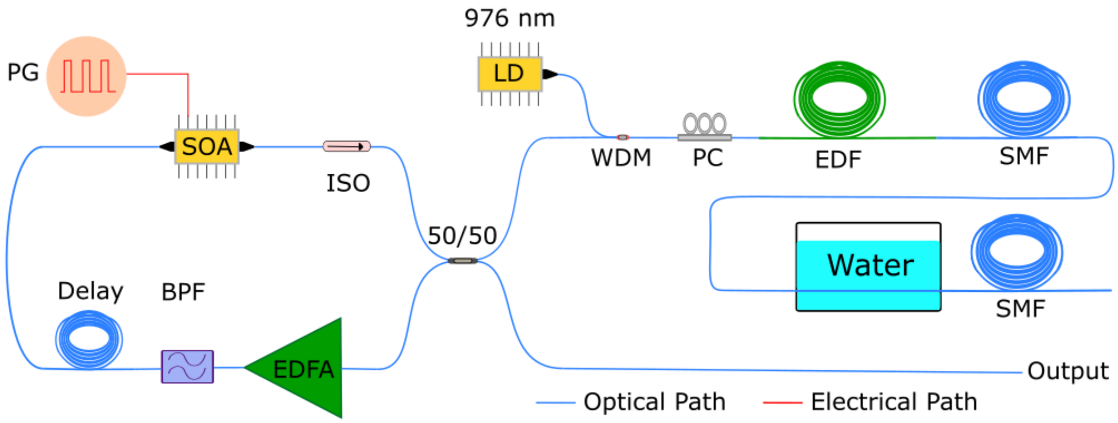

Supplementary Figure 1. **Experimental set-up**. Schematic diagram of distributed temperature sensing using Rayleigh scattering in single-mode telecom fibre. Transform-limited pulse generation makes the laser system highly spectrally efficient. Here, 9.0 cm resolution and 3.3×10^{-3}K precision were demonstrated.

The optical pulses generated by the SOA are shorter than the current pulses used for driving it, as shown in Supplementary Figure 2, for 10-ns (a) and 3-ns (b) bias. The optical pulses measured have duration 8.6 and 1.6-ns FWHM, respectively. The exact relative timing between the electrical and optical pulses is unknown. The figure shows that the response of the SOA in an open cavity (no optical feedback) and the signal-to-noise ratio is relatively poor because of the low sensitivity and high noise level of the ultrafast photodiode. Supplementary Figure 2 illustrates how sub-nanosecond optical pulses can be formed for current pulses <2 ns, and also that the shape of the optical pulse generated for the shortest durations deviates significantly from a top-hat function.

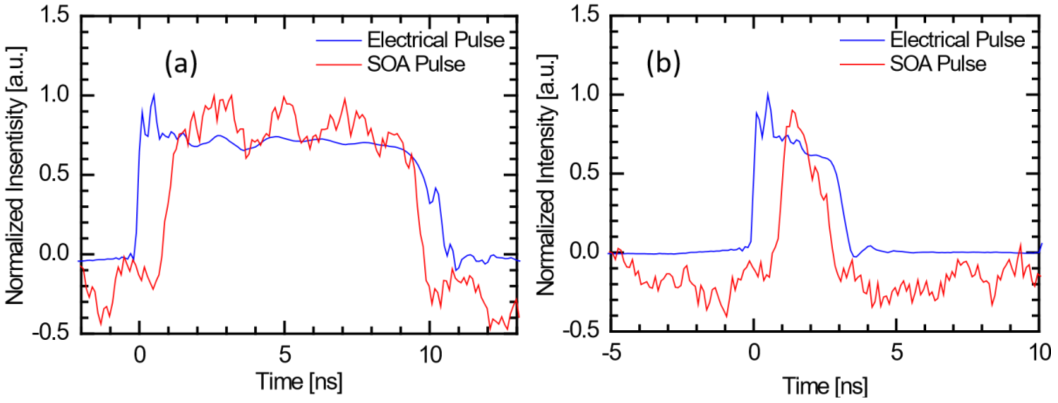

Supplementary Figure 2. **Electrical and optical pulses.** Oscilloscope traces of current pulses applied to the SOA (blue traces) and correspondent optical signals (red traces) with the optical cavity opened. (a) 10.ns current pulse; (b) 3-ns current pulse.

For completeness, Supplementary Figure 3 below shows electrical bias pulses that are replicas of the current pulses driving the SOA, and the corresponding optical pulses in a laser mode, for the spectra illustrated in Fig. 6 of the main text. The traces on the left show voltage pulses into a 50 Ω oscilloscope. The noise seen at 15 ns is due to a spurious reflection and does not appear on the optical pulses. The traces on the right display the optical pulses measured for current pulses of 5 ns, 10 ns and 20 ns. Their widths are 3.6, 8.6 and 18.7 ns, respectively, again slightly shorter than the bias pulses.

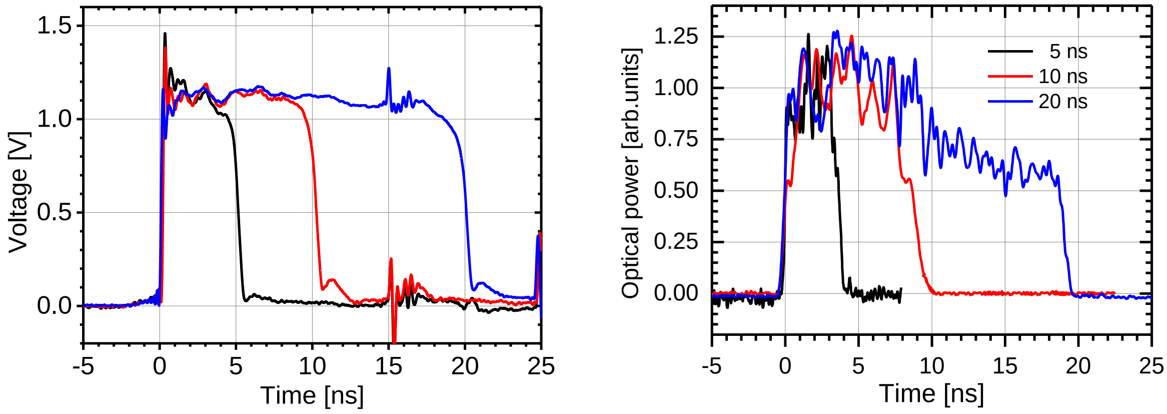

Supplementary Figure 3. **Electrical and laser pulses.** Left: replica of electrical pulses applied to SOA of 5, 10 and 20 ns; Right: Optical pulses generated, with durations 3.6 ns, 8.6 ns and 18.7 ns, respectively.

When measuring temperature, a section of the single-mode fibre was attached to the bottom of a polystyrene box containing approximately 3 litres of water. Two 4.5 digits thermometers and one thermocouple were also attached to its bottom inner surface. The voltage from the type k (ChromelAlumel) thermocouple was monitored with a microvoltmeter HP 34401A. A preliminary calibration of the thermocouple gave a linear 40 µV/K voltage dependence in the limited temperature range studied, simulating a possible dam monitoring temperature range. The resolution of the microvoltmeter and the uniformity of the temperature of the water bath were the main limiting factors in the determination of the temperature displayed in Figure 8 (main text). Increased accuracy of the measurement would require calibration of the temperature against a fixed and known value such as the fusion point of ice. Such a procedure was not carried out in this proof-of-concept experiment. The temperature of the water tank was initially raised to ~40°C and the liquid stirred. Subsequently, the polystyrene lid was closed, and the system's temperature left to gradually reduce. The cooling process took several hours. The spectral acquisition and data recording took a fraction of a minute between measured points. The range of temperatures measured was chosen to be a representative temperature at a realistic warm dam location, to allow for the temperature to be in quasi-equilibrium, and to allow for data recording before the electronics instrumentation for temperature measurements showed any variation. The error bar in the temperature measurements is ±0.0125 K. Higher precision would have been possible with better instrumentation (e.g., a nanovoltmeter to read the voltage of the immersed thermocouple).

Supplementary Figure 4 exemplifies two spectra measured of 900-ps laser pulses with the optical feedback provided by the backscattering fibre section immersed in the water tank. The blue and red traces show spectra at a temperature difference of 0.13 ^{o}C. It is clear that the entire spectrum shifts with temperature, but this shift is better quantified by determining the centre position of the laser line using a Gaussian fit, considering the 70% upper part of the signal to minimise the influence of noise. The error in the determination of the peak frequency was the standard error provided by the Levenberg-Marquardt fitting algorithm and is clearly more precise than the simple determination of the maximum, which was much more affected by the noise. The spectral peak of the two data sets can be determined with a precision ~3 MHz, corresponding to a temperature 2.5 mK. The sensitivity of the technique was evaluated by considering the quality of the least squares fit over 24 independent measurements. The average R2 value was 0.975±0.015 while the standard error in the position of the laser line was 3.2 MHz ±750 kHz, so that the overall sensitivity of a typical spectral position in this case should be ~4 MHz or 3.3 mK in this 9 cm fibre section. Indeed, a longer fibre section used to determine the average temperature would provide sharper spectral lines whose frequency could be determined with even better precision, but the spatial resolution would be compromised accordingly. This distributed temperature measurement is Fourier Transform limited: $\Delta z \times \Delta T = 3\times10^{-4}$ m.K.

It is worth stressing that the accuracy of the measurements is significantly poorer, since the two thermometers employed for absolute measurements were not calibrated against a good temperature reference. Nevertheless, in field-applications, it is often the temperature deviation from the expected value, from neighbouring points for example, daily and seasonal variations, etc., that is most useful.

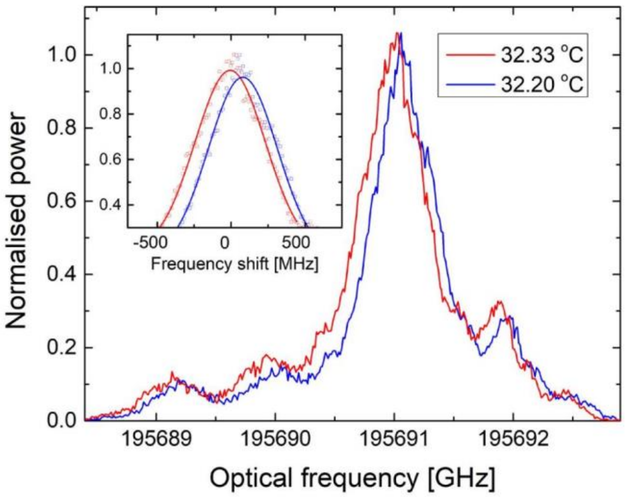

Supplementary Figure 4. **Optical Frequency Measurement.** Spectra of 900-ps optical pulses measured at close temperatures. The inset displays the Gaussian fit to the 70% upper part of the spectrum, used in the determination of the central frequency.

As discussed in the main text, mode-locking here relies on the synchronous passage of the optical pulse though a time-gated semiconductor optical amplifier, and on the spectral filtering provided by the distributed Rayleigh backscattering. Neither of these mechanisms depends on the pulse repetition frequency and one might expect that transform-limited pulses are generated for the entire roundtrip tuneability range of the laser. In order to verify this, the spectral and temporal characteristics of laser pulses generated at various frequencies were recorded and their time-bandwidth products compared. The laser pulses were measured with the instrumentation used previously. The time-bandwidth product was calculated for pulse repetition frequencies 0.7, 0.8, 0.9, 1.0, 1.1 and 1.2 MHz, covering the range studied. The pulse duration chosen was approximately 2 ns, and the pulse profile approximately square. Consistently, the power spectra of the pulses (which was collected in a single sweep without averaging) was fitted to a function $Sinc^2$. Limitations in the available electronics caused the current pulses to have some ringing and not have a perfectly flat profile (see Supplementary Figures 2 and 3). The average time-bandwidth product measured was 0.83 instead of the 0.89 expected in the ideal case of perfect $Sinc^2$ spectra and perfectly square pulses. The ~7% lower product than for the ideal case can assigned to the short pulses having a finite rise- and fall-time. The pairs of time/spectral domain measurements are included at the end of this Supplementary Information.

Supplementary Table 1. Time-bandwidth product for various laser repetition frequencies

Frequency (kHz)	Duration FWHM (ns)	Spectral width FWHM (pm)	Δν Δt Product
1203	**2.10**	**3.12**	**0.84**
1105	**2.22**	**2.91**	**0.83**
1003	**2.00**	**2.64**	**0.68**
903	**1.73**	**4.00**	**0.89**
805	**1.83**	**3.50**	**0.82**
735	**1.92**	**3.40**	**0.84**
**Average = 0.813; Standard deviation σ = 0.065;**			

Supplementary Figure 5 shows the experimental values of the time-bandwidth product measured at different laser repetition frequencies. The error bar is the standard deviation calculated for all measurements in this graph. The plot lacks a clear trend, which confirms that within the experimental error, the time-bandwidth product is independent of the repetition rate of the laser.

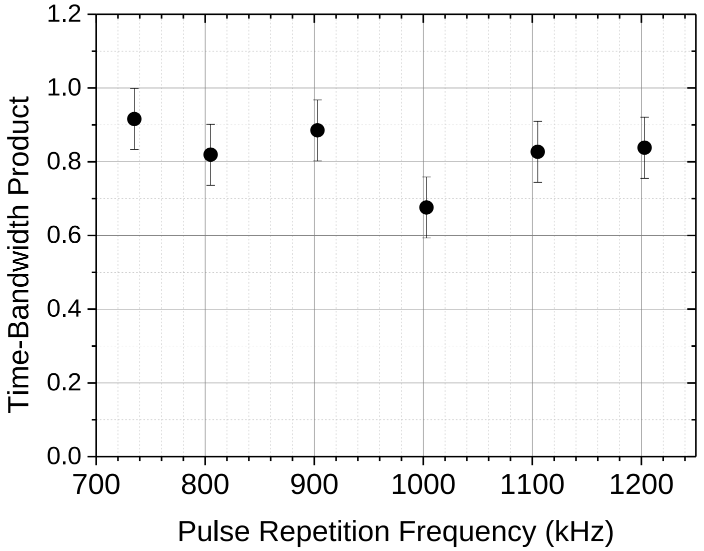

Supplementary Figure 5. **Time-bandwidth product**. Laser pulses generated at various repetition frequencies have similar time-bandwidth product, illustrating that the generation of transform-limited pulses does not depend on the repetition rate of the laser.

The present fibre laser is highly sensitive to temperature and strain variations, just as in a conventional laser with a 1-m long FBG (equivalent to the laser here with 10 ns pulses). The ability to detect 0.003 degrees shifts over 9 cm fibre segments attests to its temperature sensitivity. By immersing a section of the fibre in water inside a polystyrene ice box and choosing a part of that section as distributed backscatterer, it is possible to carry out hourly-long runs of experiments without a noticeable wavelength drift. However, long-term wavelength stability would probably require a package with active temperature stabilization. Such a package would preclude using the laser as a distributed temperature sensor in the way demonstrated in this paper and is beyond the scope of the present study.

The only means available here to keep the temperature of the laser stable in a long-term stability test is keeping the laboratory air conditioning unit at the same setting (22 ^{o}C ± 1 ^{o}C), while protecting the fibre in the cavity against air currents. A wavelength stability test was carried out over a day's work, once the temperature reached equilibrium. Only a ~2-m section of fibre, a part of which was used for backscattering, was kept under water, in the ice box used previously. Supplementary Figure 6 below illustrates a long-term measurement of the laser stability. The horizontal scale of the plot is 1 pm per minor tick, which corresponds roughly to a temperature shift of ~0.1 ^{o}C per minor division. Over the 10-h test, the wavelength of the laser drifted by less than 0.0005 nm.

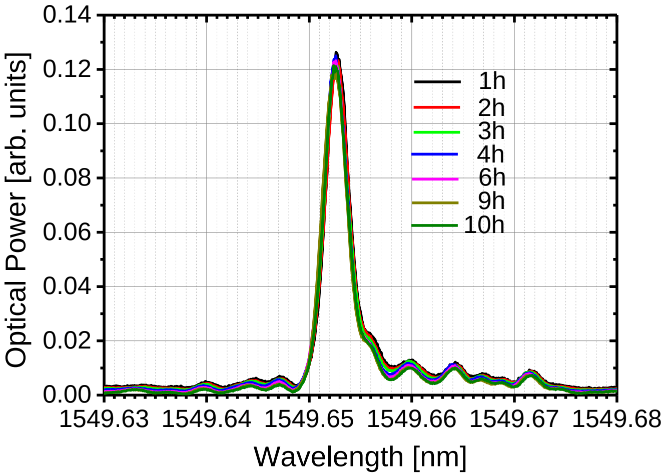

Supplementary Figure 6. **Wavelength stability test**. Long term stability test of the laser wavelength, which here drifts by less than 0.5 pm over ~10 hours.

The issue can be raised as to the role, if any, dispersion and nonlinearity play as the laser pulses travel along the fibre in the cavity. For picosecond and femtosecond pulses, the effects of nonlinearity and dispersion are usually important, but this is not the case here where the pulses are significantly longer.

The effect of nonlinearity can be estimated considering that a phase shift of π radians is induced at a length $L_{nl} = 1/\gamma P_o$, where P_o is the power of the pulse (70 mW here) and $\gamma = 2\ W^{-1}km^{-1}$ the nonlinear coefficient of SMF. The nonlinear length for calculated to be ~7 km, two orders of magnitude longer than the intracavity fibre. Likewise, the dispersion starts to play a role at the dispersion length, estimated from $L_D = T_o^2/|\beta_2|$, where the absolute value of the group velocity dispersion parameter is typically 20 ps^2km^{-1}. For the pulse durations used in this work, the dispersion length exceeds 3000 km, again orders of magnitude longer than the cavity.

One may wonder if the cavity gain equalling the loss could lead to an accumulation of dispersion/nonlinearity over successive roundtrips, but the robust mechanism for the generation of transform-limited pulses hinders such accumulation. The pulse duration is given by the current pulse applied to the SOA (e.g., 10 ns), which in turn defines the width of the random spectral peaks of the distributed mirror (e.g., ~112MHz). At every reflection, this mirror works as a strong narrow spectral filter, and the SOA as a strong time-gate. Cumulative effects are thus hindered by the filtering experienced at every roundtrip, both spectrally and temporally. The optical pulses generated cannot widen temporally in successive roundtrips because of the gating provided by the SOA, which cuts any optical emission outside the nanosecond current pulse. Likewise, it cannot widen spectrally accumulating nonlinear phase modulation because of the spectral filter that cleans the spectrum at every reflection.

Both these laser filtering characteristics can effectively limit any eventual broadening caused by dispersion or nonlinearity. Consistently, the pulses generated are measured to be transform limited.

The lack of chirp in the pulses generated by the laser here are indicative of the nonlinearity and dispersion being both negligible, or that a solitonic regime is reached where these effects cancel each other. It is hard to see how a square pulse (and not a $sech^2$) satisfies the soliton condition. Also, the photon lifetime determined from the mode bandwidth (measured to be $\Delta\nu \approx 5$ kHz in the inset of Figure 5 in the main text) is 200 µs. This corresponds to a propagation length of 40 km. This is still two orders of magnitude less than needed for dispersion to become apparent. The remaining alternative is that in the laser described and characterized here both dispersion and nonlinearity are negligible, as the calculation of L_D

and L_{NL} indicate. Once again, we attribute this to the nanosecond or sub-nanosecond pulse durations and pm bandwidths involved, a regime that differs significantly from the ps and fs regimes, where soliton effects ought to be present.

The loss of individual components and connectors and the gain provided by the amplifiers were evaluated to better understand the gain dynamics and the limits of operation of the laser. In its simplest configuration of Figure 3 (main text), dispersion shifted (DS) fibre was used. The total loss from all components splices and connections was 11 dB, including the 6-dB double pass over the coupler, whereas the maximum roundtrip-gain was found to be 69 dB, with 54.5 dB coming from the double-pass in the pumped EDF and 14.5 dB from the single-pass at the SOA. Naturally, the highest loss of the cavity was at the distributed back reflector, which amounts to the mean value of ~ -72 dB/m for a typical single-mode fibre and -69 dB/m for DS fibre. With 10-ns pulses in the DS fibre, the laser reached threshold near the maximum gain, meaning that a coherent reflectivity spike ~11 dB was available within the ~250 GHz (~2 nm) spectral region of maximum gain of the pumped EDF. Laser pulses in a standard fibre or shorter laser pulses in the DS fibre could only be observed by increasing the maximum gain, which was obtained by adding a commercial EDFA into the loop and a band pass filter to limit the ASE input on the SOA as displayed in Supplementary Figure 1. This configuration provided a maximum gain of 105 dB, which was comfortable to generate lasing pulses with durations down to ~0.3 ns, electronics limited. Furthermore, reducing the available spectral width to a flat-top 20 GHz band allowed for better stability of the laser.

Supplementary notes

Data follow with time and spectral domain measurements of laser pulses at various laser repetition rates. The time-bandwidth product for six values of the pulse repetition frequency over the range studied 0.7 – 1.2 MHz is calculated and compared, as shown in Figures 7-12. The pulse duration chosen was approximately 2 ns and the pulse profile approximately square. Consistently, the spectra of the pulses (which was collected in a single sweep without averaging) was fitted to a function $Sinc^2$. Limitations in the available electronics prevented obtaining entirely flat current pulses. The average time-bandwidth product measured was 0.83 instead of the 0.89 expected in the ideal case of perfectly square pulses and perfect $Sinc^2$ spectra (~7% difference).

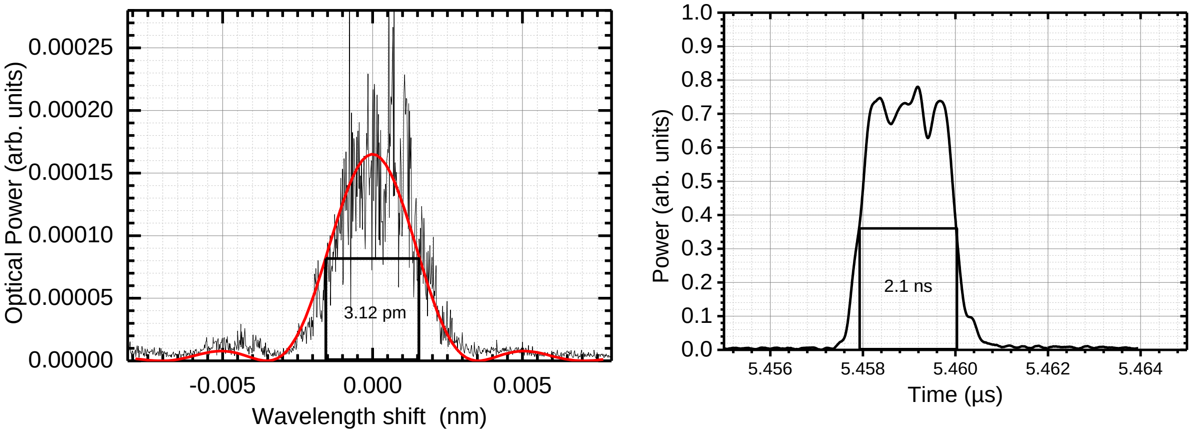

Supplementary Figure 7. **Time bandwidth product at a repetition frequency 1203 kHz**. $\Delta\lambda$ (FWHM) = 3.12 pm; $\Delta v = 3.99 \times 10^8$ Hz; Δt (FWHM) = 2.1 ns ; $\Delta v \, \Delta t = 0.838$

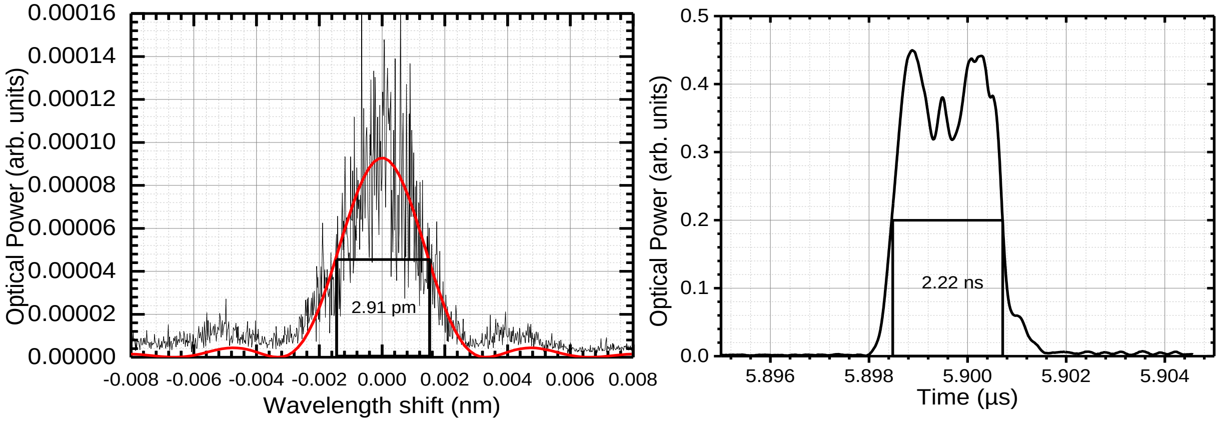

Supplementary Figure 8. **Time bandwidth product at a repetition frequency 1105 kHz**. Δλ (FWHM) = 2.91 pm; Δν = 3.72×10^{8} Hz; Δt (FWHM) = 2.22 ns; Δν Δt = 0.827

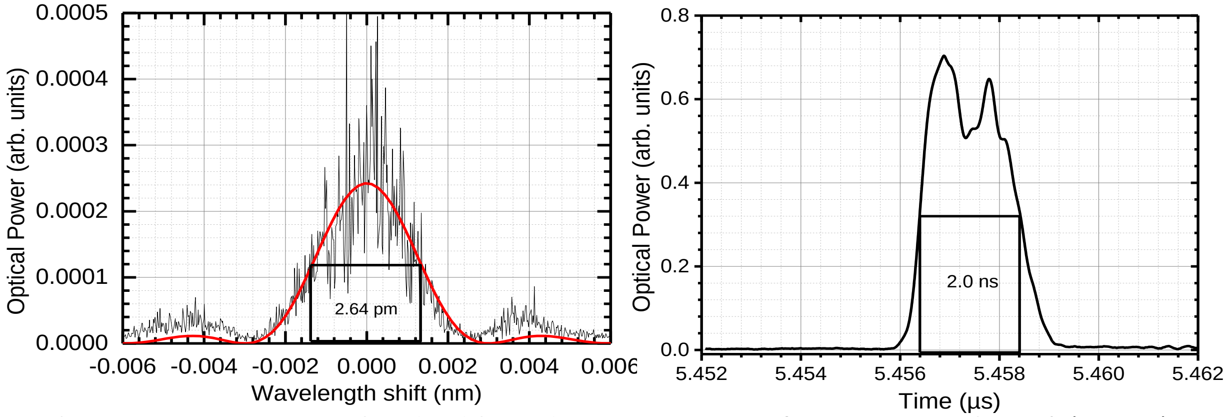

Supplementary Figure 9. **Time bandwidth product at a repetition frequency 1003 kHz**. Δλ (FWHM) = 2.64 pm; Δν = 3.38×10^{8} Hz; Δt (FWHM) = 2.0 ns; Δν Δt = 0.676

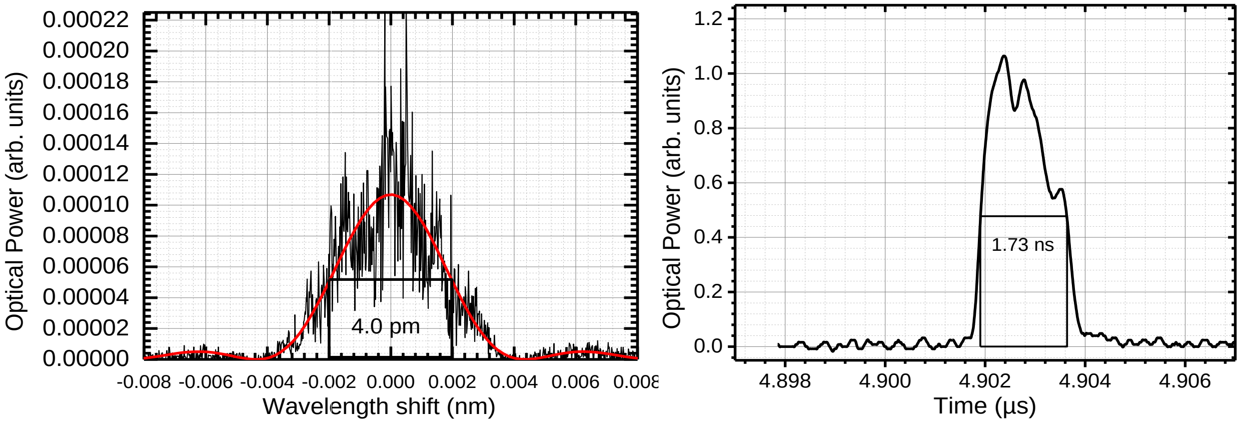

Supplementary Figure 10. **Time bandwidth product at a repetition frequency 903 kHz**.Δλ (FWHM) = 4.0 pm; Δν = 5.12×10^{8} Hz; Δt (FWHM) = 1.73 ns; Δν Δt = 0.885

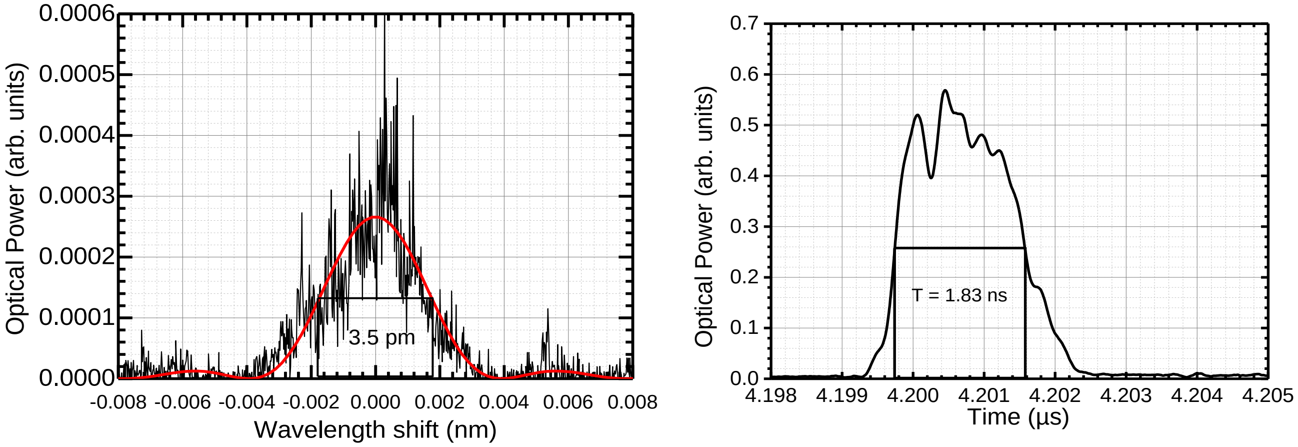

Supplementary Figure 11. **Time bandwidth product at a repetition frequency 805 kHz**. Δλ (FWHM) = 3.5 pm; Δν = 4.48x10^{8} Hz; Δt (FWHM) = 1.83 ns; Δν Δt = 0.819

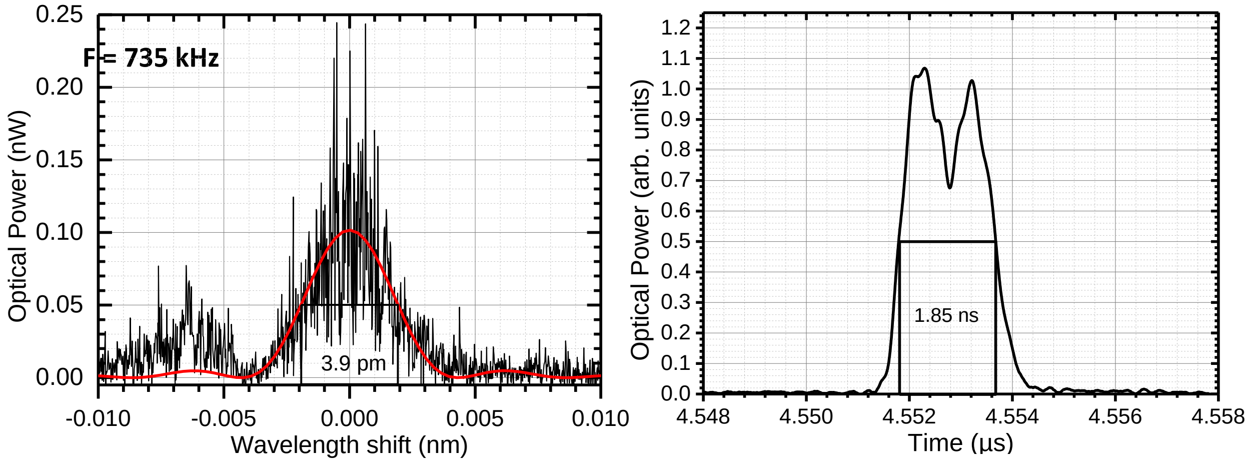

Supplementary Figure 12. **Time bandwidth product at a repetition frequency 735 kHz**. Δλ (FWHM) = 3.87 pm; Δν = 4.95x10^{8} Hz; Δt (FWHM) = 1.85 ns; Δν Δt = 0.916
